# What turns CREB on? And off? And why does it matter?

**DOI:** 10.1007/s00018-020-03525-8

**Published:** 2020-04-28

**Authors:** André Steven, Michael Friedrich, Paul Jank, Nadine Heimer, Jan Budczies, Carsten Denkert, Barbara Seliger

**Affiliations:** 1 Institute for Medical Immunology, Martin Luther University Halle-Wittenberg, Magdeburger Str. 2, 06112 Halle (Saale), Germany; 2 Institute of Pathology, Philipps University Marburg, 35043 Marburg, Germany; 3 Institute of Pathology, University Clinic Heidelberg, 69120 Heidelberg, Germany

**Keywords:** Transcription factor, CREB, Carcinogenesis, Prognosis, Clinical outcome

## Abstract

Altered expression and function of the transcription factor cyclic AMP response-binding protein (CREB) has been identified to play an important role in cancer and is associated with the overall survival and therapy response of tumor patients. This review focuses on the expression and activation of CREB under physiologic conditions and in tumors of distinct origin as well as the underlying mechanisms of CREB regulation by diverse stimuli and inhibitors. In addition, the clinical relevance of CREB is summarized, including its use as a prognostic and/or predictive marker as well as a therapeutic target.

**Electronic supplementary material:**

The online version of this article (10.1007/s00018-020-03525-8) contains supplementary material, which is available to authorized users.

## Major characteristics of CREB

Cyclic AMP (cAMP)-response element-binding protein 1 (CREB) is a 43 kDa stimulus-induced transcription factor (TF). It can bind to the cAMP response element (CRE) sequence TGACGTCA or the conserved half CRE TGACG and was first identified in the somatostatin gene promoter [1]. Genome-wide screening for CREB-binding sites suggested that more than 4000 genes might be controlled by CREB, postulating CREB as a general transcriptional activator [2].

Regarding its structure, CREB is made up of different domains with distinct functions. While the DNA binding and dimerization of CREB is mediated by a basic leucine zipper (bZIP) domain, CREB has nine serine residues in the kinase inducible domain (KID) that can be phosphorylated and activated by different kinases. Activated CREB can recruit coactivators, such as CREB-binding protein (CBP). The interaction between CREB and CBP is mediated via the interacting domain of CBP, named KIX. The CREB/CBP complex recruits the transcription machinery at the gene promoter to initiate CREB-dependent gene transcription [3]. The CREB complex upregulates the methylation of histones H3 and H4, which is essential for the initiation of the transcriptional machinery [4]. CREB activity is regulated by the phosphorylation of amino acid (aa) residues, which are mainly localized in the KID region, thereby influencing the dimerization of CREB and its binding to the CRE sequence [5]. Phosphorylation of CREB at the Ser133 residue frequently occurs, whereas phosphorylation at other serine tyrosine and threonine residues of CREB is observed at a lower frequency [5]. Interestingly, the different phosphorylation patterns of CREB are correlated with distinct cellular functions (Table 1) and can exert opposite effects: CREB^Ser111^ and CREB^Ser121^ inhibit transcription, while CREB^Ser129^ and CREB^Ser133^ induce transcription.

**Table 1:** Distinct functions of the phosphorylation sites in CREB

Serine residue	Molecular association	Induction of activity	Inhibition of activity	Cell growth	Cell mobility	Inhibition of apoptosis	Cell differentiation	Induction of transcription	Inhibition of transcription
Ser108	X								
Ser111	X								X
Ser114	X								
Ser117									
Ser121			X						X
Ser129				X				X	
Ser133		X		X	X	X	X	X	

In the following chapters, the knowledge about CREB expression, activation and clinical relevance in tumors of distinct origin and modulators of CREB that could be used as therapeutics for the treatment of diverse cancers are summarized.

## Function of CREB as a mediator of carcinogenesis: a general dogma

**Fig. 1:**
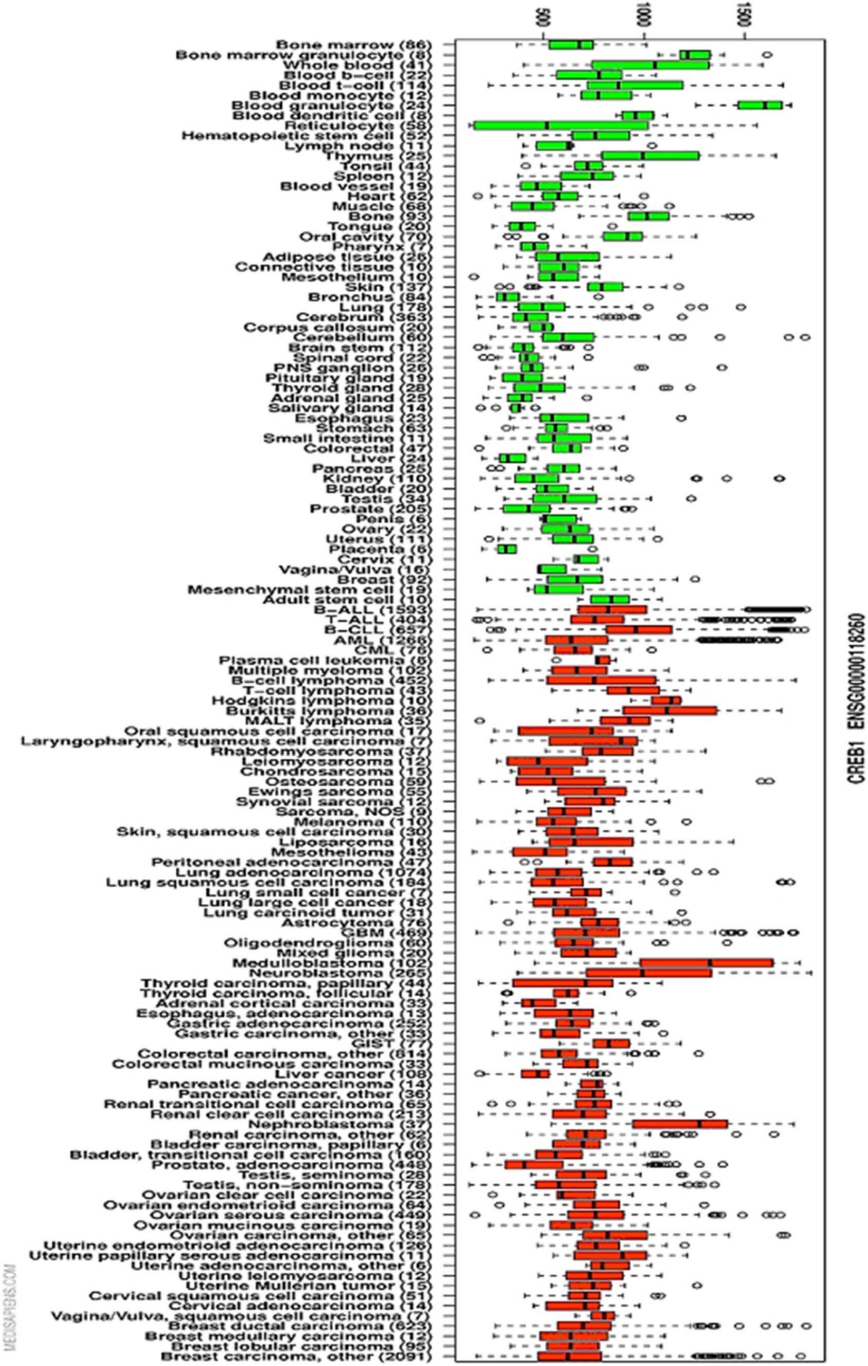
CREB expression in cancer patients. The in silico transcriptomics database (http://ist.medisapiens.com/) was employed for CREB expression in cancer and normal tissues (tissue boxplot). Green represents healthy tissue, while red represents tumor tissues

Under physiological conditions, CREB is expressed in all nucleated cells. Its expression is essential for major cellular functions, as CREB knockout mice exhibit embryonal and neuronal deficits and have a reduced lifespan [6–8]. CREB is often overexpressed in hematopoietic and solid tumors compared with control tissues, which has led to the identification of CREB-associated cancers (Fig. 1). These include acute lymphoblastic leukemia (ALL), acute myeloid leukemia (AML), Hodgkin’s lymphoma, chronic lymphatic leukemia (CLL), melanoma, hepatocellular, renal cell, ovarian, prostate, lung, gastric, esophageal, pancreatic and breast carcinoma, and brain tumors [9–11] (Supplementary Table 1).

**Fig. 2:**
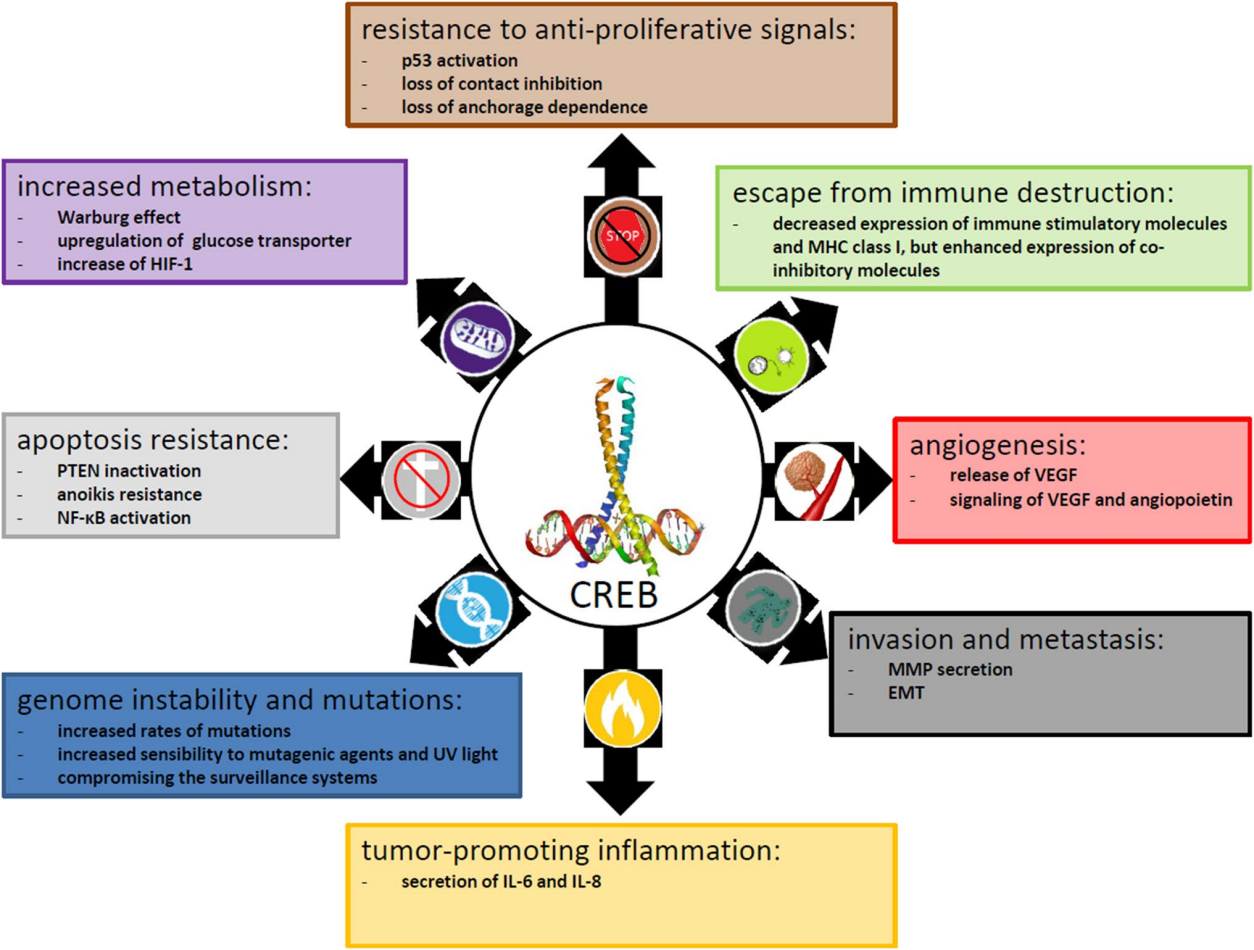
Link of the hallmarks of cancer with CREB expression and activation

In these malignancies, overexpression of CREB is associated with aberrant signal transduction caused by the deregulated expression of downstream genes that control the hallmarks of cancer, such as proliferation, apoptosis, angiogenesis, metastasis, immune surveillance, and metabolism, and the generation of tumor stem cells, which lead to the initiation and progression of tumors (Fig. 2). These different CREB activities result in increased tumor growth, resistance to antiproliferative signals, decreased apoptosis, enhanced angiogenesis, increased metabolism, and reduced immunogenicity [11–18].

## Opposing the clinical relevance of CREB in different cancers and its association with therapeutic resistance

**Fig. 3:**
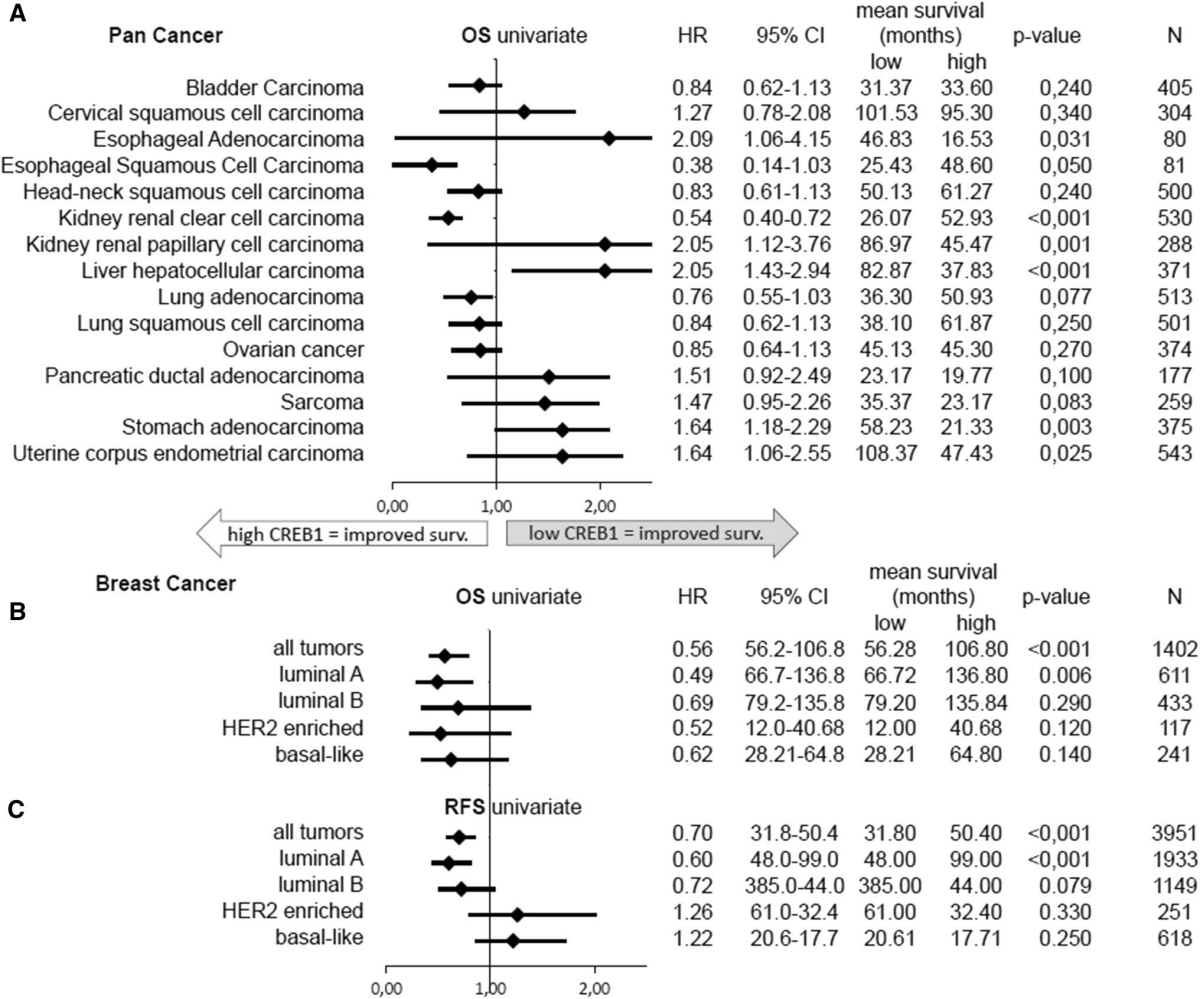
Log-rank test of continuous CREB1 expression as a prognostic marker for recurrence-free survival and overall survival. **a** Hazard ratio (HR) of overall survival from different cancer entities (pan cancer). **b** HR of overall survival from breast cancer and intrinsic subtypes. **c** HR of recurrence-free survival from breast cancer and intrinsic subtypes

In addition to the role of CREB expression and activity in different tumor entities, CREB protein levels are often correlated with clinical parameters. These include tumor grading and staging, metastasis formation, increased recurrence rates, and worse prognosis of tumor patients [19–23]. Using the KMplot mRNA gene chip and RNA-seq analysis (https://kmplot.com/analysis/), a link between CREB mRNA expression and the overall survival (OS) of patients with different tumors and tumor subtypes was reported and is summarized in Fig. 3 [24]. For example, ALL and AML patients with enhanced expression and phosphorylation of CREB at Ser133 had a decreased OS and a higher risk of tumor relapse [19, 25]. Similar data were obtained for hepatocellular carcinoma (HR 2.05, CI 1.43-2.94, *p* < 0.01), esophageal adenocarcinoma (HR 2.09, 95% CI 1.06–4.15, *p* = 0.031), and stomach adenocarcinoma (HR 1.64, 95% CI 1.18–2.29, *p* = 0.003), in which low CREB expression was associated with reduced OS (Fig. 3a). In contrast, other tumor types benefit from high CREB expression, such as clear cell renal cell carcinoma (ccRCC) (HR 0.38, 95% CI 0.14–1.03, *p* < 0.001), lung adenocarcinoma (HR 0.76, 95% CI 0.55–1.03, *p* = 0.077), esophageal squamous cell carcinoma (HR 0.38, 95% CI 0.14–1.03, *p* = 0.05), and breast cancer (BC) (HR 0.56, 95% CI 0.41–0.79, *p* < 0.001).

Regarding BC, overexpression of CREB in all intrinsic BC subtypes has been associated with improved survival of patients (Fig. 3b). In contrast, patients with HER-2/neu-positive or basal-like BC expressing high CREB levels had worse recurrence-free survival (RFS), while luminal-type A BC had an even higher RFS with enhanced CREB expression (Fig. 3c). Since CREB is often overexpressed in different tumor types, but is associated with different outcomes, the quantity of CREB (expression levels of mRNA and protein) might be less important than the quality of CREB (posttranslational modifications and dimerization). Therefore, the dual role of CREB in different tumor entities must be addressed in additional studies to obtain further insights into the underlying mechanisms of CREB regulation and function.

In addition, there is limited information about the role of CREB in therapy resistance. In BC, downregulation of CREB was associated with altered BRCA1 expression and increased expression of aromatase, a key enzyme in estrogen biosynthesis. The latter is transcriptionally regulated by CREB and associated with the development of resistance to tamoxifen [26]. Furthermore, CREB phosphorylation is involved in the development of tumor resistance to inhibitors of the MEK–ERK and PI3K/AKT pathways [26, 27]. In contrast, resistance against MAPK inhibition in BC is induced by reactivation of CREB, which is linked to an altered histone acetylation pattern [27]. In-depth characterization of the mechanisms involved in CREB-mediated therapy resistance is mandatory and relevant for improved therapeutic decision-making in tumor patients.

## Regulation of CREB activity by influencing its phosphorylation

There exists evidence that CREB activity is tightly regulated and could be either upregulated or inhibited by diverse factors. Until now, a large number of modulators known to induce CREB phosphorylation have been described in tumor cell lines and tissues of distinct origin, which include growth factors, steroid and peptide hormones, cytokines, stress, lipids, calcium and nitric oxide signaling, various viral, bacterial, and plant components, chemotherapeutics, and others (Supplementary Table 2). These stimuli exert their activities by phosphorylation of distinct CREB residues, but mainly of CREB^Ser133^, thereby affecting different signal transduction pathways, such as ERK1/2, MAPK, PI3K/AKT, CaMK, PKC, and PKA, which are known to be activated in different tumor entities.

Furthermore, high-throughput screening with compound libraries identified 1800 additional substances that were able to enhance CREB-mediated gene transcription [28]. However, most of these substances have not yet been tested in vitro and in vivo in (tumor) cell models.

In addition to stimulators of CREB activity, inhibitors of CREB phosphorylation at Ser^133^ have been identified, which might have therapeutic potential. To date, no information exists about substances negatively interfering with other CREB phosphorylation residues, as shown for the c-MET inhibitor SU11274 targeting the MET pathway [29] and for serotonin [30]. Other signal transduction inhibitors were tested in various murine and human cell systems regarding their effect on CREB expression and phosphorylation, such as H89, lapatinib, LY294002, PD98059, Ro31-8220, trametinib, the COX-2 inhibitor NS398, and the EP4 inhibitor AH23848 [31, 32]. However, these signal transduction inhibitors were not helpful for functional analysis of CREB, since they do not specifically block the phosphorylation of CREB or influence the activity of other signaling molecules. More specifically, the phosphatase-mediated dephosphorylation of CREB leads to reduced CREB activity. Most phosphatases that inactive CREB, such as protein phosphatase 2A (PP2A) [33], protein phosphatase 1 (PP1) [34], or the nuclear form of PTEN phosphatase [35], are localized to the nucleus. These phosphatases can be targeted by inhibitors, such as okadaic acid blocking both PP2A and PP1 [36]. Their deregulation is associated with altered CREB activity: under hypoxic conditions, these phosphatases are inactive, while CREB is activated and hyperphosphorylated under oxygen limitations, a condition that often occurs in the tumor microenvironment (TME).

## Other regulators of CREB expression and/or activity

### Mutations in the CREB gene

Structural alterations of CREB have been reported in tumor cell lines and lesions of distinct origin, but their frequency is highly varied. These alterations are diverse and include amplification, homozygous deletions, missense, and in-frame and truncated mutations and fusions, as summarized in Supplementary Tables 3 and 4. Mutations of the CREB gene are most common in neuroendocrine prostate cancer and cervical carcinoma. Missense mutations or truncated mutations have been found, but are not associated with the function of CREB. Furthermore, amplification of the CREB gene has been reported in many tumors. The structural abnormalities of CREB are often accompanied by alterations of other genes known to be involved in tumorigenicity.

A number of studies described gene rearrangements of the Ewing’s sarcoma (EWS) gene with CREB1 in various rare diseases, such as clear cell sarcoma (CCS), CCS associated with the gastrointestinal tract, and angiomatoid fibrous histiocytoma [37–40]. EWS/CREB fusion in CCS associated with the gastrointestinal tract resulted in the loss of KID and was accompanied by melanin pigmentation of these tumors [37]. This is caused by low expression levels of genes involved in melanogenesis, such as MITF and TYR, representing an indicator of deregulated CREB activity. In addition, gene fusions were reported for the CREB family members ATF1 and CREM, particularly in tumors of young children [39], in rare cases of angiomyeloid fibrous histiocytoma [41] and in myxoid neoplasms [40].

### Regulation by epigenetic modification, such as methylation and histone modification

There exists only limited information on the epigenetic control of CREB. By employing TF arrays, CREB was identified among 42 TFs to interact with the DNA methyltransferases DNMT3A and DNMT3B [42]. In addition, there is an epigenetic modification switch mediated by the CRE element. After methylation of the central CpG, binding of CREB and related TFs to CpMetG is not possible [43, 44], while binding of the TF C/EBPα is promoted [45], resulting in the control of tissue-specific gene expression [46]. A well-studied example is the CRE site in the BRCA1 gene [47]. CREB is considered a positive regulator of BRCA1, since the methylation of CpG islands significantly reduces its expression. A similar relationship has been reported for MMP-13 [48], which is only transcribed upon demethylation. Nuclear magnetic resonance studies suggest that methylation affects the flexibility of DNA, thereby reducing the ability of TFs to bind to DNA [49].

### microRNAs

Posttranscriptional regulation is of crucial importance for the control of gene expression and is mainly mediated by the interaction of RNA-binding proteins (RBPs) and/or microRNAs (miRNAs) with the 3′-untranslated region (3′-UTR) of the respective gene [50–53]. Discordant CREB mRNA and protein expression has been found in some tumor cells, suggesting the regulation of CREB at the posttranscriptional level. Furthermore, it is noteworthy that the annotated ~ 9.000-nucleotide-long 3′-UTR of CREB (ENST00000432329.2) is well above average (~ 800 nucleotides) [54] and gives rise to extensive regulation via this region. In silico analysis and CLIP data revealed a number of well-characterized RBPs potentially binding to CREB, e.g., FUS/TLS (Fused in Sarcoma/Translated in Sarcoma) and RBM10 (RNA-binding protein motif 10) [55, 56]. However, to the best of our knowledge, there is no proven interaction between RBPs and CREB, illustrating the need for further research. In contrast, a number of CREB-regulating and CREB-regulated miRNAs have been recently described in tumor cell lines and in tumors of distinct origin, which are summarized in Table 2 [57].

**Table 2:** Characterization of CREB-regulating (RC) miRNAs or CREB-regulated (CR) miRNAs in human tumors and tissues or cell lines

Name	Cell line/tumor	CR, RC miRNAs	References
miR-181b	Gastric cancer	RC	[132]
miR-34b	AML	RC	[60]
miR-200b	Astrocytoma	RC	[133]
miR-181a	PC12 (pheochromocytoma)	RC	[134]
miR-9	Glioblastoma	RC, CR	[61]
miR-433-3p	Glioblastoma	RC	[135]
miR-372	Liver cancer	RC	[136]
miR-1271	Prostate cancer	RC	[137]
miR-760	Colorectal cancer	RC	[138]
miR-23a	Glioma	CR	[139]
miR-27b	HepaRG liver cells	CR, RC	[223]

In leukemia, the CREB protein is overexpressed, which is associated with a poor outcome in these patients [58, 59]. Pigazzi and coworkers demonstrated that miR-34b is involved in the oncogenesis of various tumors and is a major regulator of CREB expression. A direct interaction of this miRNA with the 3′-UTR of CREB was described. In AML, the miR-34b/-34c promoter is hypermethylated and provides a mechanism for the low miR-34b expression in this disease [60].

However, particularly in the context of a general TF such as CREB, it is obvious that miRNA-dependent deregulation is more than a one-to-one relationship. For example, a regulatory mechanism was reported for miR-9 and CREB, whereby CREB promotes the transcriptional expression of miR-9, and in turn, miR-9 directly targets the 3′-UTR of CREB. The balance between these two players is supposed to coordinate the migration and proliferation potential of glioma cells, which may help cells adapt rapidly to environmental changes [61]. Furthermore, miR-27b targeted CREB, demonstrating a positive correlation between CREB and miR-27b in gastric cancer, suggests a bidirectional CREB–miR-27b interaction. This hypothesis is supported by the presence of several CREB-binding sites in the putative promoter of miR-27b [62]. Thus, a better understanding of the CREB–miRNA regulatory networks may open new perspectives for novel therapeutic targets in human malignancies.

### Posttranslational modifications with the exception of phosphorylation

It is generally accepted that posttranslational modifications (PTMs), such as acetylation, phosphorylation, glycosylation, SUMOylation, and ubiquitination, often occur (Supplementary Fig. 1) and are altered during physiologic and pathophysiologic cellular processes. Furthermore, these PTMs were also found for CREB and were associated either with increased or decreased CREB activity, which was mediated by distinct mechanisms, as summarized in Table 3. Several PTMs of CREB can affect the progression of cancer and have been recently extensively reviewed [63].

**Table 3:** Different PTMs of CREB and their functional relevance

Modification	aa residue in CREB	CREB activity	Mechanism	Species	References
Acetylation	K136	Increased	Recruitment of CBP/p300	M, 3T3-L	[140]
	K136	Increased	Deacetylation by SirT1	H, HEK293T	[141]
	K91, 94, 136	Decreased^a^	Acetylation by CBP/p300	R, F9; Mo, COS-7	[142]
	n/a	Increased	HDAC9 regulating CREB mRNA	H, HuH7	[143]
	K136	Increased	CREB acetylation increased by low glucose	M, hippocampal cells	[144]
Ubiquitination	K48-linked^b^	Decreased	TRAF3 increasing ubiquitination	M, B cells	[145]
	n/a	Decreased	MTUS1 deubiquitinating CREB	H, THP-1 cells	[146]
	K48-linked^c^	Decreased	Hypoxia-mediated ubiquitination	M, NIH3T3 cells	[147]
	n/a	Decreased	H_2_O_2_-induced ubiquitination	D, in vivo	[148]
	n/a^c^	Decreased	PDGF-stimulated phosphorylation of S103/S107	R, pulmonary artery	[149]
	n/a	Decreased	Hypoxia-mediated loss of PP1 activity	H, CaCo-2 cells	[150]
	n/a	Decreased	Hypoxia-mediated ubiquitination	H, HeLa; BT, T84	[151]
SUMOylation	K271, K290	Increased	PIAS1-induced modification with SUMO-1	H, HEK293T	[152]
	K285, K304	Increased	Hypoxia mediated by SUMO-1	H, HeLa; BT, T84	[151]
	K285, K304	Increased	Hypoxia mediated by SUMO-1,2,3	M, NIH3T3 cells	[147]
O glycosylation	S40, T228	Decreased	Elevated CRTC/TORC interaction	R, neuronal cells	[153]
	n/a	Decreased	Nuclear import under high glucose	H, HuH7	[154]
	T256, S260	Decreased	Disrupted interaction with TAFII130	R, brain	[155]
	n/a	Decreased	Iron-induced decreased levels of O-GlcNAcylated	M, 3T3-L	[156]
phosphorylation (not in KID)	S270/S271	Decreased	DNA damage	H, HeLa; H, HEK293T	[157, 158]
	S271	Increased	Genotoxic stress	H, SH-SY5Y; H K562	[158]

Species: *M* mouse, *H* human, *R* rat, *D* dog, *BT* cow, *Mo* monkey;* n/a* not analyzed

^a^Triple mutants only; in single mutants, no changes were observed; enhanced CREB-mediated gene expression, when inhibition of histone deacetylase activity by trichostatin A

^b^Polyubiquitinated chain, CREB aa not assigned

^c^Polyubiquitinated chain and monoubiquitination, presumably CREB-K330 or K339

### Dimer formation of CREB (homodimers and heterodimers)

The dimer formation of CREB has been controversially discussed. For example, CREB dimerization with ATF1 was described in HeLa cells, but these heterodimers had a lower stability and CRE binding activity than the CREB homodimers [64]. Furthermore, the CREB:ATF1 heterodimers were predominantly found in undifferentiated cells, while homodimer formation was mainly detected in differentiated cells [65, 66]. Regarding jun/fos, CREB:fos heterodimers exist, but their formation is ineffective [67]. In contrast, Muchardt and coauthors reported that neither jun nor fos form heterodimers with CREB, suggesting cell-specific control of this process [68]. In line with these data, no ATF1:jun or ATF1:fos heterodimers could be detected, but heterodimer formation between ATF4 and jun/fos occurred [69]. However, dimer formation of CREB with other bZip TFs has not yet been analyzed in detail in different tumor entities.

### Localization-dependent activity of CREB

Under physiological conditions, CREB is localized in the nucleus, while under pathophysiological conditions, e.g., in a hypoxic microenvironment, CREB is shuttled to the mitochondrial matrix [57], where it binds to the mitochondrial CRE sequence. This process results in the control of mitochondrial gene transcription [70], which can be blocked by H89. These data suggest a localization-dependent activity of CREB. Chalovich and coauthors demonstrated that the equilibrium between nuclear and cytoplasmic CREB can be triggered to the site of cytoplasmic localization by 6-hydroxydopamine (and therefore enhancing the levels of mitochondrial CREB) [71, 72]. While Cammarota and coworkers localized phosphorylated CREB in the mitochondria [73], the antibody reacts with an epitope of mitochondrial pyruvate dehydrogenase, suggesting a non-CREB-specific signal [74]. In more recent studies, different CREB-specific antibodies directed against different epitopes of the non-phosphorylated form, gel shift assays [57, 72, 75] or ^35^S-methionine-labeled CREB have been applied, demonstrating that CREB could be localized in mitochondria under certain conditions [70]. In addition, irradiation can increase the amount of CREB^Ser131^ in the nucleus, which might represent a resistance mechanism of prostate cancer cells [21]. Furthermore, the quantity and activity of the CREB protein in the nucleus can be increased by high glucose levels [76], which are often associated with enhanced tumor cell metabolism, calcium influx [77], or thrombin [78].

## Experimental modulation of CREB expression and/or activity

### Molecular approaches by CREB silencing

In addition to chemical compounds, diverse experimental approaches, e.g., shCREB, siCREB, double negative (DN) CREB, and CRISPR/CAS, have been used to downregulate or inhibit CREB expression. Although CREB protein expression can be transiently repressed by siRNA binding to CREB1 mRNA [79, 80], long-term experiments exceeding 96 h were not possible. Therefore, shRNA constructs against CREB1 have been commonly used for analyses of the long-term effects of CREB [32, 81]. The specificity of these constructs was proven by monitoring the expression of CREB-related ATF1 and CREM. The implementation of a dominant negative construct as well as reconstitution of CREB knockdown is necessary to rule out unspecific effects. Different dominant negative forms of CREB to block its expression or activity were developed, including a construct named A-CREB, in which the bZIP domain was replaced with an acid amphipathic sequence [82]. This construct mimics the polarity of the CRE sequence and can form a heterodimeric complex with CREB, resulting in decreased CREB binding to the CRE sequence. Another dominant negative form of CREB is the overexpression of a mutated CREB protein, which contains a KID with a replaced amino acid. Furthermore, CREB^Ser133^ has been mutated to CREB^Ala133^, which prevents CREB phosphorylation at this position [83, 228]. A similar approach has been employed for the inhibition of phosphorylation at other serine residues [84]. Furthermore, the DNA-binding domain has also been mutated [83], while Aucoin and coworkers (2004) used double-negative forms of CREB to efficiently block the invasion potential of melanoma cells [226]. Dominant CREB repressors were successfully used both in vitro and in vivo [85], resulting in increased oxidative stress in a transgenic mouse model. In this context, it is noteworthy that silencing or deleting CREB by, e.g., CRISPR/Cas-9, has not been successfully established [86], since CREB is critical for the survival of cells. CREB knockout is lethal in mice, as CREB knockout causes deficits in embryonal development [6, 87]. To circumvent cell death mediated by CREB knockout, the generation of inducible constructs is suggested. Interestingly, the CREB-mediated transduction of cAMP signaling and CREB function in vivo could be partially compensated by CREM [7].

### Chemical compounds

#### Small molecule inhibitors

**Fig. 4:**
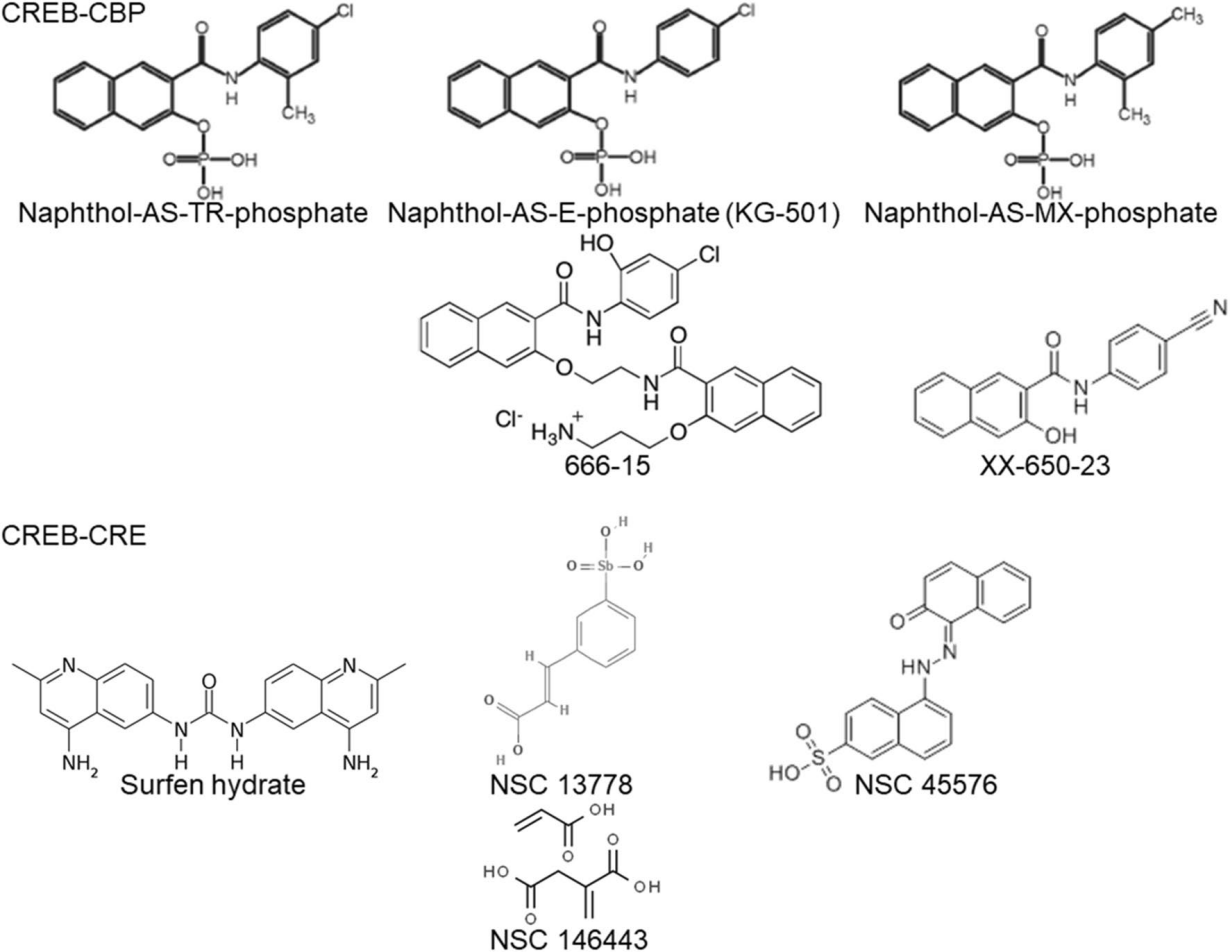
Chemical structure of CREB-specific small molecule inhibitors. NSC 146443 is a 1:1 mixture of 2-methylenesuccinic acid and acrylic acid and can form a polymer (Pubchem CID: 161509)

Two different strategies are currently used to block CREB activity with high specificity using chemicals/inhibitors. Based on nuclear magnetic resonance (NMR) analysis demonstrating the binding of CREB KID to CBP KIX [88–90], the interaction between CREB and the coactivator CBP was targeted using CREB-CBP inhibitors, such as different naphthol derivatives [91] (Fig. 4). Furthermore, the binding of CREB to the CRE-DNA element can be blocked with substances binding to the DNA major groove (positively loaded substances) or directly to the bZIP of the TF (negatively loaded substances). A live imaging system using a bioluminescence-based detector system for the analysis of the interaction of KID and KIX was developed by Ishimoto and coworkers [92], which enables screening for CREB inhibitors, e.g., in herbal extracts [93].

#### CREB-CBP inhibitors targeting the KID-KIX interaction

Various CREB-CBP inhibitors exhibit distinct activities, which are summarized in Supplementary Table 5. The CREB-CBP inhibitor naphthol-AS-E-phosphate (KG-501) was identified by Best and coworkers [94] based on molecular modeling for chemicals binding to the pocket of the KID domain, and was the first inhibitor used for this blocking mechanism in cell culture [95]. KG-501 is specific for CREB, as it blocks the interaction between KID and KIX only at the CREB-specific binding site of KIX, named the c-myb site [96], where the nonphosphorylated proteins c-myb, p53, and BRCA1 can bind [97, 98]. The other binding site of KIX (MLL), where MLL, c-jun, and HIV-1 TAT can bind, is blocked by pamoic acid (KG-122). However, CREB Ser133 had the highest affinity for the c-myb site (Kd CREB − CBP = 700 nM; Kd c-myb − CBP = 15 µM, Kd p53 − CBP > 90 µM) [99]. The unphosphorylated form as a physiological inhibitor was analyzed in different tumor cells using an FRET-based test system. In ALL cell lines, this inhibitor caused increased apoptosis [25], while it blocked CREB phosphorylation induced by curculigoside A and diminished tube formation [100]. In HER-2/neu-overexpressing cell lines, KG-501 decreased migration and anchorage-independent growth without influencing CREB expression and phosphorylation [32]. Due to the relatively low potency of this inhibitor (Ki ~ 90 µM) and its reduced solubility, different structural analogs have been synthesized in recent years [91, 101–103], such as naphthol-MX-phosphate and naphthol-AS-TR-phosphate. However, MX-phosphate is less efficient than KG-501 (IC50 9.7 µM vs 6.9 µM), while AS-TR-phosphate is more potent, as decreased anchorage-independent growth and cyclin expression were detected at lower concentrations (IC50 3.7 µM) [104].

The 3-(3-aminopropoxy)-*N*-[2-[[3-[[(4-chloro-2-hydroxyphenyl)amino]carbonyl]-2-naphthalenyl]-oxy]ethyl]-2-naphthalenecarboxamide hydrochloride inhibitor (666-15) is an improved, highly efficient CREB-CBP inhibitor (IC50 ~ 80 nM) [94, 167] that weakly affects NF-κB activity by blocking the CBP–NF–κB interaction (IC50 5290 nM). In vivo experimental murine studies of 666-15 revealed its quick bioavailability; no effects were found on kidney and heart functions [105], and it was, therefore, well tolerated in the mouse model. The synthesis of this chemical was first described by Xie et al. [183], followed by the synthesis of different regioisomers [106]. Some modifications for higher aqueous solubility were recently introduced to the backbone of this inhibitor, and results showed that 666-15 had a higher IC50 but inhibitor combinations conferred an additional effect [107]. In a murine xenograph model, 666-15 suppressed the tumor growth [183].

The *N*-(4-cyanophenyl)-3-hydroxy-2-naphthamide inhibitor (XX-650-23) was synthesized by Li et al. and Xie et al. [101, 103]. XX-650-23 blocks the interaction of CREB and CBP in AML cells expressing high CREB levels, leading to cell cycle arrest and apoptosis by activating caspase-3 activity and decreasing the expression of the antiapoptotic CREB-regulated BCL-2 protein [108]. XX-650-23 is more efficient than KG-501 (IC50 ~ 3 µM in a luciferase detection system). AML cells with higher CREB protein expression, such as HL-60 cells, had an IC50 < 1000 nM, while the CREB low-expressing MOLM-13 cells had an IC50 > 2000 nM. Thus, a specific inhibitory potential seems to be possible for patients with higher CREB levels. Indeed, patients with primary AML or relapsed AML showed higher CREB expression than healthy individuals, and treatment of bone marrow with 2 µM XX-650-23 for 48 h increased the number of dead cells in AML samples but not in normal bone marrow cells [108]. Niclosamide, a molluscicide, had similar effects to XX-650-23 on CREB activity and viability of AML cell lines, but lacks the naphthalene ring [109]. As shown in a recent study further modifications of the XX-650-23 compound lead to better physiological stability and improve the potency [230]. *N*-(4-Chlorophenyl)-3-hydroxy-2-naphthamide is a cell permeable naphthamide compound that directly binds to the KIX of CBP with an IC50 < 3 µM. It blocks firefly luciferase activity (IC50 ~ 1 µM) but not Renilla luciferase activity.

#### CREB-CRE inhibitors targeting the interaction of CREB and DNA

Different CREB-CRE inhibitors have also been developed, but are currently less frequently used than KID-KIX inhibitors. These include Surfen (Surfen hydrate, Alias: NSC 12155; CAS-No: 3811-56-1), which is commonly used as a disinfection agent in wound healing solutions or as a depot in combination with insulin, but was withdrawn due to strong allergic reactions. Surfen is an antagonist for heparan sulfate [110], and its potential CREB-CRE blocking mechanism was described by Rishi and coworkers (2005), who reported that Surfen has a higher specificity for CREB than for C/EBPβ (EC50 0.6 µM vs. 2.5 µM) [112]. Surfen at lower concentrations has been shown to block the binding of CREB to a CRE oligonucleotide, accompanied by reduced proliferation of BC cell lines [111].

Stibavirin (Alias: NSC 13778; CAS-No: not registered) is an arylstibonic acid that was proven to bind the basic leucine zipper of CREB but not to DNA [112]. It is, therefore, a specific inhibitor for CREB but also for fos/junD (EC50 13.9 vs. 2.5) [113] and binds to CD4^+^ T cells [114]. Furthermore, NSC 13778 blocks the binding of TFE3 type 1/2 to the specific promoter element [115], while its derivative P6981 had a stronger effect on CREB inhibition [116]. However, neither substance is commercially available.

The inhibitor 5-[(2-hydroxy-1-naphthalenyl)azo]-2-naphthalenesulfonic acid (Alias: NSC 45576; CAS-No: 68133-05-1) has been proposed as a therapeutic agent for AML, because it decreases the proliferation of AML cell lines. Furthermore, NSC45576 influences cAMP/PKA signaling by reducing the activity of PKA holoenzymes [117, 118].

## CREB, ATF2, and c-jun: “It stays in the family”

CREB is a member of the bZIP TF family consisting of approximately 20 ATF/CREB family members [119, 120]. Since many TFs can bind to CREB-binding elements, the analysis of whether CREB can be replaced by other TFs is crucial for targeted therapies. Studies have revealed that knocking down CREB expression or activity significantly decreases the transcription of many CRE-regulated genes, such as bcl-2 [18], suggesting that CREB is the major regulator of these genes. Furthermore, genes with a nonpalindromic CRE regulator element, e.g., a half CRE element such as TCAGC, are often downregulated in CREB deficiency and sometimes more efficient than full CRE genes. This could be explained by a stronger induction of CREB at a half CRE sequence than at a full CRE sequence [68] due to a higher competition to members of the CREB-ATF family at the complete CRE site. Interestingly, the activity of the full CRE sequence is higher than that of half CRE sequences in the absence of CREB [68]. Therefore, it is likely that ATF1 can partially compensate for the loss of CREB activity, which is limited due to the lower stability of ATF1 and CREM homo- and heterodimers [3].

**Fig. 5:**
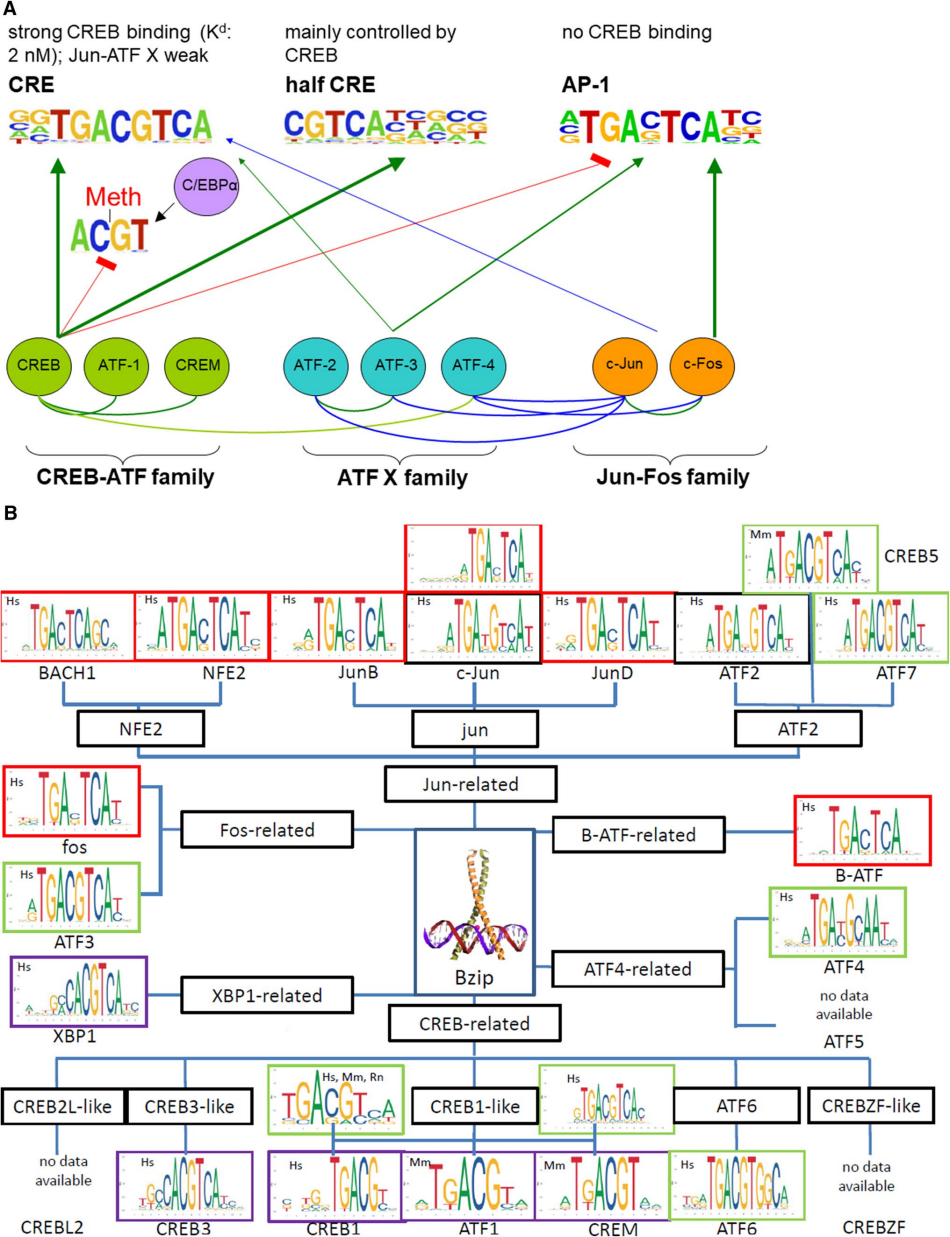
Model for heterodimerization and the interaction of CREB and other bZIP TFs with DNA elements. **a** Shown are three bZIP TF families (CREB-ATF, ATF X, and jun-fos) with representative examples. Possible heterodimerization processes are displayed by curved lines: dark green lines represent intrafamily heterodimerization, dark blue lines represent heterodimerization between ATF X and the jun-fos family, and bright green lines represents (rare) heterodimerization between the CREB-ATF and ATF X family. The arrows show the binding of homo- and heterodimers at CRE, half CRE, and AP1 sequences; the line thickness represents the binding affinity, and the line color represents the specificity of the complexes. Jun and fos can bind only as heterodimers with ATF X on a CRE element (blue arrow). CREB-ATF cannot interact with a CpG-methylated CRE or AP1 sequence (red arrows), but C/EBPα can bind to a methylated CRE (black arrow). The conservation of binding sequences was taken from the HOMER database (http://homer.ucsd.edu/homer/motif/motifDatabase.html). **b** The conservation sequence of the DNA-binding motifs for different bZIP TFs was taken from the JASPAR2020 database (http://jaspar.genereg.net/). The bZIP TFs were classified according to the entrance into the TFClass library (http://tfclass.bioinf.med.uni-goettingen.de/). Common bZIP TFs are presented. The colored boxes around the sequences are as follows: green = typical 8 bp full CRE sequence with a central conserved CG (TGACGTCG), violet = half site CRE (TGACG), red = typical 7 bp AP-1 site without a central CG (TGACTCA or TGAGTCA), and black = 8 bp binding site without a conserved central CG. The species are abbreviated as follows: Hs = *Homo sapiens*, Mm = *Mus musculus*, and Rn = *Rattus norvegicus*. The central bZIP motif is the CREB1 leucine zipper domain binding to the CRE-DNA and was taken from the PyMOL data bank PDB1DH3

Other bZIP TFs, such as ATF2 or ATF3, which cannot form heterodimers with CREB or ATF1 [119], can also bind to the CRE element. They can form heterodimers with jun and fos, and allow binding to the CRE element but with a lesser affinity than CREB. Experiments performed by Hai and Curran [69] revealed that jun/fos heterodimers with ATF2/3 can bind to CRE but not to half CRE. Furthermore, jun and fos heterodimers can bind with higher affinity to AP-1 and full CRE sequences compared to half CRE sites [225]. Therefore, CREB competes with the heterodimers jun-fos/ATF2/3 at CRE but not at half CRE, as summarized in Fig. 5a, b.

Furthermore, CREB prefers the central CpG dinucleotide of CRE/half CRE, which explains the lack of CREB binding to the CRE-similar AP1 sequence [121]. A methylated CpG dinucleotide is a binding site for C/EBPα but not for CREB [46]. Genes with a CRE element and a TATA box could show different regulation than CRE genes without a TATA box. Binding of CREB to the 8 bp CRE sequence and the variable half CREs depends on different mechanisms [122]. Exclusively, CREB can bind to two different half CRE motifs with a dissociation constant that is comparable to that of the full CRE sequence [68, 119, 123].

In addition to ATF1 and CREM, the cAMP response transcriptional coactivators (CRTCs), comprising the three members CRTC1, CRTC2, and CRTC3, represent an additional family of CREB coactivators with similar modular structures. CRTCs are evolutionarily highly conserved and sequestered in the cytoplasm [124]. They have been shown to regulate transcriptional activation and pre-mRNA splicing via distinct functional domains [125]. CRTCs upregulate the activity of CREB by association with residues in the bZIP domain. However, CRTCs not only regulate CREB-dependent target genes but also CREB-independent transcriptional responses. The aberrant activation of CRTCs in tumors is linked with oncogenic activities, such as migration, invasion, and metastasis formation, representing all hallmarks of cancer [126, 127]. This is also strengthened by the fact that mutations in CRTCs have been shown to be key drivers in the development and progression of cancer [128].

## Conclusion: CREB as a prognostic biomarker or therapeutic target?

Based on the central role of CREB in the initiation, maintenance, and progression of many cancer types (Supplementary Fig. 2), CREB is considered a prognostic biomarker and an excellent therapeutic target structure for tumors. This claim is supported by expression analyses of the early, inducible cAMP repressor (ICER), an inhibitor of CREB, which is downregulated in BM cells of AML patients [60]. An advantage of using CREB as a target structure is its ability to regulate different signal transduction pathways, which are often aberrantly activated in tumors. However, it is noteworthy that high CREB expression in some tumor types is associated with better patient outcomes. To date, the underlying mechanisms of these opposing effects are not well understood and require further investigation.

Several strategies are currently used to inhibit CREB function in tumor cells: (1) Initial studies focused on dominant negative CREB mutants (KCREB) to block CREB transcription. KCREB cannot bind to CRE sequences but forms heterodimers with wild-type CREB. Overexpression of KCREB in metastatic tumor cells decreases the metastatic potential in vitro and in vivo [129]. (2) CREB decoy oligonucleotides that efficiently inhibit CREB-mediated gene transcription and therefore negatively influence tumor growth have been developed [130]. (3) CREB expression is silenced by RNA interference, which not only modulates cell viability and growth properties but also enhances apoptosis. shRNA-mediated silencing of CREB expression is coupled with diminished growth of tumor cells, increased apoptosis, cell cycle arrest in the G0/G1 phase and suppression of anchorage-independent growth [32, 61, 131].

Since these proof-of-principle studies have revealed therapeutic effects, alternative strategies to inhibit CREB-mediated gene transcription with small molecule inhibitors have been developed. For example, kinase inhibitors can prevent phosphorylation and, therefore, inhibit the activation of CREB. In addition, chemical inhibitors can block the interaction of CREB-CRE or CREB-CBP [91, 94, 101, 103]. Naphthol-AS-E-phosphate (KG-501) reversibly and dose-dependently disrupts the interaction between the KID domain of CREB and the KIX domain of CBP but not forskolin-stimulated phosphorylation at Ser133. Micromolar concentrations of KG-501 can modulate the cAMP-dependent expression of CREB target genes without off-target inhibition. Another strategy is the modulation of CREB-regulating miRNAs [22]. Since CREB has many oncogenic properties and participates in the induction of resistance mechanisms, it is a promising target for the treatment of many tumor types; nevertheless, for tumor types in which high levels of CREB expression are associated with better outcomes, this approach may not be suitable.

### Electronic supplementary material

Below is the link to the electronic supplementary material.

Supplementary material 1 (PDF 464 kb)

## Data Availability

All data are available upon request from the corresponding author.

## Abbreviations

ALL: Acute lymphatic leukemia
AML: Acute myeloid leukemia
ATF-1: Activating transcription factor 1
BC: Breast cancer
bZIP: Basic leucine zipper
CaMK: Calcium-activated calmodulin kinase
CBP: CREB-binding protein
CLL: Chronic lymphatic leukemia
CRE: cAMP response element
CREB: cAMP response element-binding protein
CREM: cAMP response element modulator
CRTC: cAMP response transcriptional co-activator
DNMT: DNA methyltransferase
ERK: Extracellular signal-regulated kinase
EWS: Ewing’s sarcoma
HR: Hazard ratio
KID: Kinase-inducible domain
KIX: KID-interacting domain
MAPK: Mitogen-activated protein kinase
MFS: Metastasis-free survival
miRNA: microRNA
OS: Overall survival
PI3K: Phosphatidylinositol 3-kinase
PK: Protein kinase
PP1: Protein phosphatase 1
PP2A: Protein phosphatase 2A
PTM: Post-translational modification
RBP: RNA-binding protein
RCC: Renal cell cancer
RFS: Recurrence-free survival
TCGA: The Cancer Genome Atlas
TF: Transcription factor
TME: Tumor microenvironment
TNBC: Triple negative breast cancer
UTR: Untranslated region
