# Supplementary material for: What turns CREB on? And off? And why does it matter?
Source: Cell Mol Life Sci. 2020 Apr 28;77(20):4049–67. doi: 10.1007/s00018-020-03525-8 (PMC7532970; doi:10.1007/s00018-020-03525-8)
Supplement: Supplementary file 1 — Supplementary material 1 (PDF 464 kb) [file 18_2020_3525_MOESM1_ESM.pdf]

What turns CREB on? And off? And why does it matter?

Cellular and Molecular Life Sciences

André Steven[1], Michael Friedrich[1], Paul Jank[2], Nadine Heimer[1], Jan Budczies[3], Carsten Denkert[2], Barbara Seliger[1]

[1] Institute for Medical Immunology, Martin Luther University Halle-Wittenberg, 06112 Halle (Saale), Germany

[2] Institute of Pathology, Philipps University Marburg, 35043 Marburg, Germany

[3] Institute of Pathology, University Clinic Heidelberg, 69120 Heidelberg, Germany

Corresponding author:	Prof. Dr. Barbara Seliger
	Martin Luther University Halle-Wittenberg
	Institute for Medical Immunology
	Magdeburger Str. 2
	06112 Halle (Saale), Germany
Telephone:	(+49) (345) 557 - 1357
Fax:	(+49) (345) 557 - 4055
E-mail:	barbara.seliger@uk-halle.de

Note: The listed references in this document are included under their indicated number in the main manuscript.

Supplementary Material

Supplementary Tables

Supplementary Table 1: Modulation of CREB protein in tumors and its clinical relevance. Phrases in italics reflect outcomes that differ from the majority of the literature.

Carcinoma	CREB expression in cancer vs normal	Clinical relevance and prognosis	References
ALL	CREB overexpression	n/a	[159]
	p-CREB overexpression	poorer survival	[25]
AML	CREB overexpression	less favorable prognosis	[19]
breast cancer	increased (p-)CREB	poor prognosis, metastasis, nodal involvement	[160]
	increased CREB	n/a	[20]
	increased (p-)CREB	n/a	[32]
colon cancer	increased p-CREB	n/a	[161]
	increased (p-)CREB	n/a	[131]
ESCC	CREB overexpression	correlation with lymph node metastasis and tumor-node-metastasis (TNM) stage	[9]
gastric cancer	increased p-CREB	n/a	[162]
		poor survival	[163]
glioblastoma/glioma	increased p-CREB	poor survival	[164]
	increased CREB	promotes proliferation	[139]
	CREB overexpression	shorter OS and PFS	[61]
	CREB overexpression	correlation with tumor grading	[133]
	CREB overexpression	correlation with tumor grading, poorer survival	[18]
hepatocellular carcinoma	increased CREB	n/a	[165]
	increased CREB	high CREB leads to shorter DFS and OS	[166]
	increased (p-)CREB	high (p)CREB leads to shorter DFS and OS	[167]
Hodgkin’s lymphoma	*decreased CREB*	*high expression of CREB correlates with favorable prognosis*	[168]
kidney, renal cell carcinoma, clear cell	increased (p-)CREB	CREB increased migration and invasion	[15]
	increased CREB	correlation with higher TNM stages	[169]
laryngeal cancer	CREB overexpression	association with cancer differentiation, tumor stage, and lymphatic metastasis	[17]
leukemia, lymphatic	CREB overexpression	poor outcome	[170]

leukemia, myeloid	CREB overexpression	n/a<br>induction of aberrant myelopoiesis	[171]<br>[172]
lung cancer	increased (p-)CREB<br>increased p-CREB/CREB	CREB is significantly upregulated<br>overexpression of CREB or p-CREB was related to a lower probability of survival.	[173]<br>[23]
medulloblastoma	increased p-CREB	*p-CREB favorable prognosis*	[229]
meningioma	increased p-CREB	correlation with angiogenesis + recurrence	[174]
NSCLC	increased p-CREB	*favorable prognosis for smokers and squamous cell carcinoma*	[175]
prostate cancer	increased p-CREB and p-CREB/CREB ratio	n/a	[176]
ovarian cancer	increased CREB	promotes cell proliferation	[177]
SCLC small cell lines carcinoma	increased p-CREB	promotes cell proliferation	[11]
skin cancer, malignant melanoma	increased p-CREB<br>increased p-CREB<br>increased p-CREB	melanoma progression<br>tumor growth and metastasis<br>increased p-CREB correlates with metastasis	[178]<br>[179]<br>[180]
thyroid cancer	CREB overexpression	n/a	[181]

n/a = not analyzed

Supplementary Table 2: Known stimuli modulating CREB activity and downstream signal pathways in human und murine tumor cells.

Stimulus	CREB residue	Protein kinases	Cell model, tissue	References
Growth factor signaling/kinases				
c-KIT/MC1R	n/a	PKA, ERK	melanoma cells, h	[182]
EGF	Ser133	ERK1/2, AKT	IGROV1 cells, h (ovarian cancer)	[183] [219]
HER-2/neu receptor	n/a	n/a	metastatic breast cancer tissue, h	[184]
Serum	Ser133	ERK 1/2, p38	Caco-2 cells, h	[185]
Steroid hormone signaling				
17β estradiol	Ser133	n/a	TNBC cells, h	[186]
Calcitriol	n/a	PI3K/AKT	HeLa cells, THP-1 cells, h	[187]
Corticosteroids	n/a	n/a	4B cells, h (hypothalamic cell line)	[188]
Peptide (hormone) signaling				
Catecholamine	Total CREB	cAMP/PKC	SKOV3 cells, h (ovarian cancer)	[189]
Cytokines				
IL-1β	Ser133	ERK1/2	gastric cancer, h	[224]
NO and oxidative stress				
ER stress	Ser131	n/a	MDA-MB231, h (breast cancer)	[190]
H_2O_2	Ser133	PKA, ERK	C10 cells, h (colorectal adenocarcinoma)	[191]
H_2O_2	Ser121	n/a	K562 cells, h (myelogenous leukemia), L-40 (lymphoblasts)	[192]
Hypoxia	Ser133	PKA	T cell lymphoma, m	[193]
Hypoxia	Ser131	n/a	MDA-MB231, h (breast cancer)	[190]
Viral, bacterial and plant components				
Acacetin	n/a	ERK1/2	B16F10 cells, m (melanoma)	[194]
Angelica sinensis polysaccharides	n/a	ROCK1	T47D, Hs578T, h (breast cancer)	[195]

Diosmetrin	n/a	ERK1/2	B16F10 cells, m (melanoma)	[194]
Sulforaphene	Ser133	MSK1	Eca109, h (esophageal cancer)	[196]
Yessotoxin	Ser133	mTOR	leukemia cells, h	[197]
Phospholipids and lipid signaling				
placental total lipid	Ser133	p38 MAPK	B10F10 cells, m (melanoma)	[198]
Environmental stress factors				
DNA damage	Ser121	ATM	HEK293T, MeWo, HELA, h	[199]
FCS depletion	Ser133	JNK	HCT116, h (colon cancer)	[200]
Glucose deprivation	Ser121	n/a	U2OS cells, h (osteosarcoma)	[201]
Glucose deprivation	Ser129	GSK3α	PC12 cells, r (pheochromocytoma), F9 cells (teratocarcinoma)	[202]
IR	Ser108, 111, 114	n/a	HEK293T, MeWo, HELA, h	[199] [203]
UVB	Ser133	p38	SKM1(acute myeloid leukemia), *in vivo*, m	[204]
Ion channels and intracellular Ca^{2+} signaling				
KCl	Ser142	nuclear CaMK II	PC12 cells, r (pheochromocytoma)	[205]
Chemotherapeutics				
4-Hydroxytamoxifen	n/a	AKT	MCF-7, SKBR-3, h (breast cancer)	[206]
Doxorubicin	Ser133	ERK	malignant mesothelial, h	[207]
"genotoxic stress"/DNA damage	Ser270	HIPK2	K562 cells, h (myelogenous leukemia), SH-SY5Y, h (neuroblastoma cells)	[158]
Quinaldic acid	Ser133	AKT	HT-29, LS180. Caco-2, h (colorectal carcinoma)	[208]
Retinoic acid	Ser133	n/a	neuroblastoma cells, h	[209]

n/a = not specified; Species: m = mouse, h = human, r = rat

Supplementary Table 3: Structural alterations of the CREB1 gene in different tumor entities.

Tumor entity	n	Genetic alterations (order)	Frequency of mutation rate [%]	Study
bladder cancer	408	deep deletion > amplification	1.5	TCGA, [210]
breast invasive carcinoma	996	amplification > deep deletion > missense mutation = fusion	1.3	TCGA, panCancer Atlas
cervical squamous cell carcinoma and endocervical adenocarcinoma	191	deep deletion > missense mutation	4.0	TCGA, provisional
esophageal adenocarcinoma	265	amplification	3.0	TCGA, [211]
esophageal carcinoma	184	amplification > deep deletion	2.7	TCGA, provisional
head and neck squamous cell carcinoma	504	deep deletion > amplification > truncating mutation	1.6	TCGA, provisional
kidney renal clear cell carcinoma	448	amplification > deep deletion	1.3	TCGA, provisional
kidney renal papillary cell carcinoma	280	deep deletion > amplification = missense mutation	1.4	TCGA, provisional
lung adenocarcinoma	230	amplification > missense mutation	1.7	TCGA, [212]
lung squamous cell carcinoma	469	amplification > deep deletion = missense mutation	1.3	TCGA, panCancer Atlas
metastatic prostate adenocarcinoma	444	amplification	4.0	[213]
neuroendocrine prostate cancer	114	amplification	12	[214]
ovarian serous cystadenocarcinoma	311	amplification > deep deletion	5.0	TCGA, provisional
pan-lung cancer	1144	amplification > missense mutation > deep deletion > in-frame mutation	1.3	[215]
prostate adenocarcinoma	1013	amplification > missense mutation > deep deletion	1.8	[216]
stomach adenocarcinoma	393	amplification > missense mutation > deep deletion > truncating mutation	3.0	TCGA, provisional
uterine corpus endometrial carcinoma	242	missense mutation > amplification = deep deletion = truncating mutation	2.9	TCGA, provisional
uveal melanoma	80	deep deletion	1.3	TCGA, panCancer Atlas

n = number of samples

The cBioPortal database (https://cbioportal.org/) was used for the analysis of the mutation load in different tumors. The study with the highest number of samples was chosen. Only tumor entities with a mutation rate > 1.0% and > 50 samples were included. The most common genetic alterations are listed first.

Supplementary Table 4: Mutation rate of CREB1 in different tumor cell lines.

Cell line	Tumor entity	CREB alterations	Other notable mutations
Capan-1	mixed, ductal adenocarcinoma from liver metastasis	amplification	ERBB2 amplification, MYC amplification, KRAS (G12V), TP53 (A159V)
J82	bladder cancer, transitional cell carcinoma	amplification	PTEN deep deletion, BCL2L1 amplification, TP53 (E271K)
MJ	cutaneous T cell lymphoma	amplification	MYC amplification, ERBB4 amplification, MYB (Y629H)
MOTN-1	T cell leukemia, T cell large granular lymphocytic leukemia	amplification	MYC amplification, BRCA1 amplification
NCI-H661	lung cancer, large cell carcinoma	amplification	KRAS amplification, CCNE1 amplification, TP53 (R158L, S215I)
OVKATE	ovarian cancer, adenocarcinoma	amplification	KRAS amplification, CCND1 amplification, TP53 (R282W)
RD	rhabdomyosarcoma	amplification	BRCA2 deep deletion, MYC amplification, NRAS (Q61H), TP53 (R248W)
ALLSIL	hematopoietic and lymphoid tissue mixed cancer types	deep deletion (homodeleted)	TP53 deep deletion, NOTCH1 (L1593P)
EC-GI-10	esophagus carcinoma	deep deletion (homodeleted)	ERBB2 amplification, MYC amplification, TP53 (Y234C, R273L)
ME1	acute myeloid leukemia, hematopoietic and lymphoid tissue	deep deletion (homodeleted)	TP53 deep deletion, CCND1 amplification, NRAS (Q61H)
MPP 89	mesothelioma	deep deletion (homodeleted)	TP53 deep deletion, ATM deep deletion, RB1 (V654M)
OCI-LY19	B cell lymphoma	deep deletion (homo deleted)	MYC amplification, FGFR1 amplification, NRAS (Q61K), CREBBP (D1435E)
OVCAR-8	ovarian cancer, adenocarcinoma	deep deletion (homozygous deleted)	MYC amplification, B2m deep deletion, ERBB2 (G776V), TP53 (X126_splice)
BT474	breast cancer, ductal carcinoma	truncating mutation (Y252*)	ERBB2 amplification, CCND1 amplification, BRCA2 (S3094*), TP53 (E285K)
KCL-22	chronic myeloid leukemia in blast crisis, Philadelphia chromosome-positive CML	truncating mutation (R95Tfs*14)	USP6 deep deletion, PIK3CA(E545G), CREBBP (Q2045*)
HCC70	breast cancer, ductal carcinoma	missense mutation (R298Q)	TERT amplification, RICTOR amplification, PTEN (F90Lfs*9), TP53 (R248Q)
LoVo	colorectal cancer, large intestine	missense mutation (P75Q)	DUSP22 deep deletion, B2m deep deletion, KRAS (G13D), APC (R1114*)
MDA-MB-453	breast cancer, ductal carcinoma	missense mutation (E319K)	ERBB2 amplification, MYC amplification, PIK3CA(H1047R), PTEN (E307K)
SNU175	colorectal cancer, large intestine, adenocarcinoma	missense mutation (T324A)	ERCC2 deep deletion, SUFU deep deletion, KRAS (A59T), EGFR (A864V)
SW1116	colorectal cancer, large intestine, adenocarcinoma	missense mutation (L234V)	KRAS (G12A), TP53 (A159D), SMAD2 deep deletion

For the analysis, CREB-mutated tumor cell lines were screened in cBioPortal with the dataset “Cancer Cell Line Encyclopedia (Novartis/Broad, Nature 2012)”

Supplementary Table 5: Small molecule inhibitors targeting the interaction between CREB and CBP (KID – KIX) and their *in vitro* and/or *in vivo use*.

Inhibitor	Cas-No.	Cancer entity/cell line	Used concentration & incubation time	Reference
Naphthol-AS-E-phosphate (KG-501)	18228-17-6	ALL pheochromocytoma	10 – 50 µM, 1 -2 d	[25]
		A549 (lung adenocarcinoma)	1 – 10 µM, n/a	[101]
		PC12 (pheochromocytoma)	10 µM, 72 h	[217]
		U-87MG (glioblastoma)	10 µM, 72 h	[218]
		HBMEC	25 µM, 4 h	[100]
		HER-2/neu^{+} BC, KRASV12	25 µM, 24 h	[32, 131]
		Human lung cancer	1 – 10 µM, 24 h	[104]
		NSCLC/HUVEC	5 – 20 µM, 96 h	[95]
		NSCLC/HUVEC	10 µM, 24 h	[95]
Naphthol-AS-MX-phosphate	1596-56-1	Human lung cancer cells	5 – 20 µM, 96 h	[104]
Naphthol-AS-TR-phosphate	2616-72-0	Human lung cancer cells	5 – 20 µM, 24 h/96 h	[104]
3-(3-Aminopropoxy)-N-[2-[[3-[[(4-chloro-2-hydroxyphenyl)amino]carbonyl]-2-naphthalenyl]oxy]ethyl]-2-naphthalenecarboxamide hydrochloride (666-15)	1433286-70-4	BC cells	1 nM – 1 µM, 72 h	[219] [111]
		MDSC	100 nM, n/a	[220]
		Neuroblastoma	5 µM, 12 h	[132]
		PDAC, *in vivo* (mouse)	10 mg/kg BW/d for 3 weeks	[221]
		in vivo (mouse)	10 mg/kg BW/d (5 times a week over three weeks)	[105]
		N2A cells (neuroblastoma)	5 µM, 12 h	[227]
N-(4-cyanophenyl)-3-hydroxy-2-naphthamide (XX-650-23)	117739-40-9	AML cell lines	0.01 – 10 µM, 48 h	[108]
		AML cell lines	0.1 – 10 µM, 48 h	[109]
		in vivo (mouse)	2 mg/kg (single dosis)	[230]
N-(4-Chlorophenyl)-3-hydroxy-2-naphthamide (Luciferase inhibitor III)	92-78-4	HEK293T	10 – 100 µM, n/a	[94]
		HEK293T	1 – 100 µM, 2 – 4.5 h	[222]

The different inhibitors were tested on different murine or human cell lines at varying concentrations and for different time points. n/a = not specified

Supplementary Figures

Supplementary Figure 1:

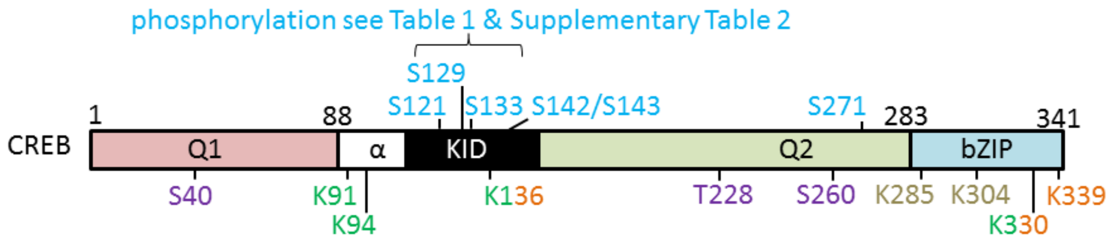

Acetylation
O glycosylation
Phosphorylation
Ubiquitination
SUMOylation

Supplementary Figure 2:

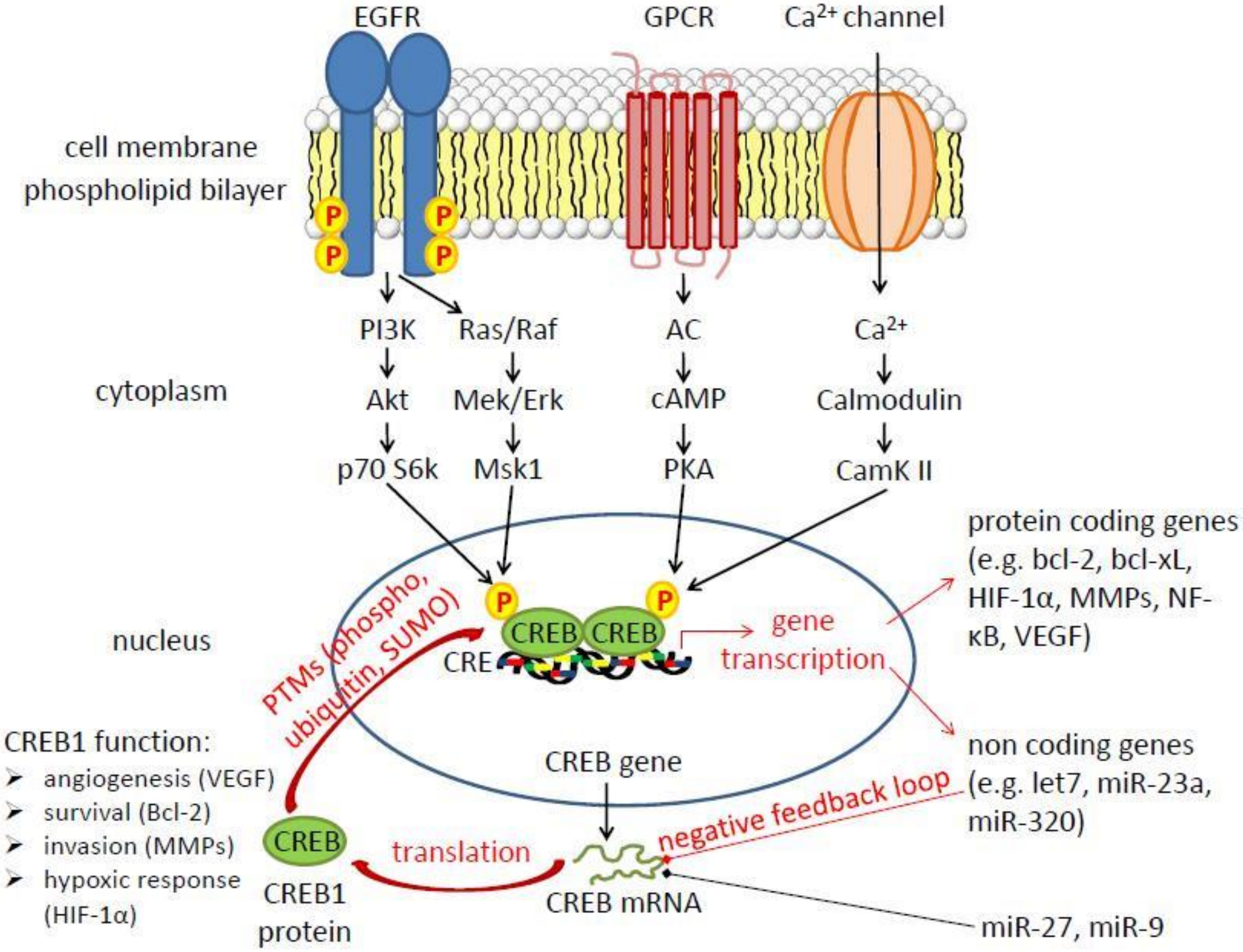

Supplementary figure legends

Supplementary Figure 1: Domain structure of CREB and important aa residues.

The scheme shows the longest CREB1 protein isoform with 341 aa. Important amino acid residues that can be posttranslational modified are numbered. Most serine residues that can be phosphorylated (blue) are localized in the KID but also in the glutamine rich Q2 domain. Ubiquitination (orange) or SUMOylation (brown) is possible through lysine site-chains in the bZIP, while lysine connected with acetylation (green) are mainly found in the KID and the α region. O glycosylation (purple) is possible in the Q2 domain.

Supplementary Figure 2: CREB is a central player in gene regulation.

The transcription factor CREB is of central importance in oncogenesis. Several signal transduction pathways (e.g., PI3K/AKT, RAS/MEK, cAMP/PKA) may lead to activation of CREB phosphorylation, causing dimerization of CREB and binding to the CRE-DNA element. On the one hand, CREB regulates protein-coding genes, such as bcl-2, as well as noncoding genes of miRNAs or long noncoding RNAs. In the latter case, a negative feedback loop is also possible because some of the CREB-regulated miRNAs themselves can target CREB mRNA. Furthermore, posttranslational modifications of the CREB protein can significantly influence its activity. In addition to the abovementioned phosphorylation, this also includes modifications by ubiquitination or SUMOylation. The activity and function of CREB, such as the promotion of angiogenesis, are thus influenced not only by the expression level but also by the PTMs of CREB.
